# Functional Hydrogels in Food Applications: A Review of Crosslinking Technologies, Encapsulation Trends, and Emerging Challenges

**DOI:** 10.3390/polym17212955

**Published:** 2025-11-06

**Authors:** Sebastián Catalán Briones, Cassamo U. Mussagy, Fabiane O. Farias, Andrés Córdova

**Affiliations:** 1 Escuela de Alimentos, Pontificia Universidad Católica de Valparaíso, Waddington 716, Playa Ancha, Valparaíso 2360100, Chile; scatalanbiones@gmail.com; 2 Escuela de Agronomía, Pontificia Universidad Católica de Valparaíso, Quillota 2260000, Chile; cassamo.mussagy@pucv.cl; 3 Department of Chemical Engineering, Polytechnique Center, Federal University of Paraná, Curitiba 81531-990, PR, Brazil; fabianefarias@ufpr.br

**Keywords:** hydrogels, crosslinking mechanisms, bioactive encapsulation, food applications, biopolymers, controlled release

## Abstract

Hydrogels derived from natural and synthetic polymers have emerged as versatile materials with wide applications in food science, biotechnology, and health-related sectors, providing unique opportunities to encapsulate, protect, and deliver bioactive compounds, as well as to create new textures and functional properties in food systems. This review summarizes the latest advances in the design and application of hydrogels, highlighting the critical relationship between polymer structure, crosslinking strategies, and functional performance. The analysis reveals that while significant progress has been achieved, challenges persist in scaling laboratory-scale hydrogel systems to industrially relevant processes, where stability, reproducibility, and regulatory acceptance remain major bottlenecks. Emerging directions in the field include the development of smart hydrogels that respond to environmental stimuli (pH, temperature, or enzymatic activity), sustainable fabrication routes using renewable biopolymers, integration with advanced processing technologies such as 3D printing or microfluidics, and biorefinery approaches emphasizing their role in valorizing agro-industrial by-products into high-value functional materials. Hydrogels represent a promising platform at the interface of polymer science, food technology, and biotechnology, whose continued development will depend on multidisciplinary innovation aiming to meet consumer demands for sustainable, safe, and health-promoting food systems.

## 1. Introduction

Hydrogels are biomaterials with a highly hydrated three-dimensional structure, formed by polymeric networks capable of absorbing and retaining large amounts of water without dissolving. This capacity confers unique properties that have aroused great interest in disciplines such as medicine, biotechnology, materials science, and the food industry [1,2].

Thanks to their molecular architecture based on hydrophilic domains (-OH, -COOH, -NH_2_), hydrogels can create stable aqueous microenvironments, similar to the biological extracellular matrix. This similarity favors their integration into living systems, in addition to granting them characteristics such as elasticity, viscoelasticity, and responsiveness to external stimuli [3,4]. From a structural standpoint, hydrogels can be formed from natural polymers (proteins, polysaccharides), synthetic (PEG, PVA) or hybrid polymers, and can be crosslinked through chemical, physical, or enzymatic mechanisms [5], which allows modulation of their mechanical, rheological, and controlled release properties [2,6].

In the food sector, these polymeric systems are positioned as emerging platforms for encapsulation and controlled release of bioactive compounds. Their application allows protection of sensitive nutrients such as vitamins, probiotics, and antioxidants against adverse conditions during processing, storage, or digestion, improving their stability and bioavailability [7,8,9]. Likewise, the possibility of co-encapsulating multiple functional agents has led to formulations aimed at personalized nutrition, aligning with current consumption trends. Nevertheless, technological challenges persist, such as incompatibility with lipophilic molecules or degradation in gastric environments, which limit their industrial scalability [10].

The aim of this review is to provide a comprehensive and critical overview of hydrogel crosslinking mechanisms—including chemical, physical, enzymatic, and ultrasound-assisted approaches—with special emphasis on their functionality, biocompatibility, and applicability in foods. Particular attention is given to their potential for encapsulation and delivery of bioactive compounds, recent trends and industrial scalability. For such purposes, the manuscript is organized into the following core sections: (i) crosslinking mechanisms, (ii) structural and functional properties, (iii) applications in foods, (iv) current industrial challenges, and (v) emerging trends in hydrogel design. With all the above, this review integrates updated findings with comparative analysis, offering guidance for researchers and food product developers working in innovations.

## 2. Classification According to Origin

Hydrogels can be classified according to the origin of the polymers that compose them into three major groups: natural, synthetic, and hybrid. This classification directly influences their physicochemical properties, biocompatibility, biodegradability, and specific applications.

### 2.1. Natural Hydrogels

Natural hydrogels are composed of biopolymers such as alginate, chitosan, gelatin, agarose, and collagen. These materials have garnered significant scientific interest due to their distinctive characteristic properties, including biodegradability, biocompatibility, hydrophilicity, superabsorbency, and viscoelasticity [6]. Their chemical structure favors cell adhesion and their ability to mimic the extracellular matrix, making them particularly attractive candidates for biomedical and food applications [11].

The biodegradable nature of numerous hydrogels enables their cross-linked chains to undergo degradation through the action of enzymes, microorganisms, humus, and water molecules [2], thereby facilitating their enzymatic breakdown in biological environments. However, these advantageous characteristics also present significant drawbacks, as the weak mechanical properties and facile degradation constitute major limitations that restrict their practical applications [2]. Additionally, these materials face challenges in terms of reproducibility, mechanical strength, and stability under variable environmental conditions.

### 2.2. Synthetic Hydrogels

Polymers designed in laboratory settings serve as the foundation for synthetic hydrogels, with polyethylene glycol (PEG), polyacrylamide (PAAm), and poly(N-isopropylacrylamide) (PNIPAAm) representing commonly employed examples. The appeal of these materials lies in their capacity to provide precise manipulation of network architecture, chemical makeup, and mechanical behavior, attributes that render them particularly suitable for contexts demanding stability, resistance, and reproducibility [2]. The formation of chemical crosslinks within these polymer networks enables several advantageous characteristics, notably substantial water sorption capacity, reproducibility in fabrication, and improved physical and chemical stability, alongside enhanced gel strength [11].

Among the synthetic polymers utilized in hydrogel development, PEG merits particular attention due to its distinctive properties. This polymer demonstrates solubility in aqueous media, exhibits biocompatibility, and undergoes biodegradation. A significant aspect of PEG’s utility stems from its capability to form conjugates with peptides, proteins, and certain pharmaceutical compounds. Beyond these applications, PEG finds use in grafting processes with natural polymers, thereby introducing specific properties necessary for targeted applications [6]. However, appropriate modifications remain essential to ensure adequate biocompatibility of these synthetic materials.

### 2.3. Hybrid Hydrogels

Hybrid hydrogels combine natural and synthetic polymers to leverage the advantages of both types. The rationale behind this approach stems from complementary limitations: biopolymer-based matrices excel in compatibility and degradability but lack structural robustness and exhibit premature release kinetics, whereas synthetic counterparts deliver mechanical stability at the expense of biological performance. Integration of both components yields composite architectures with expanded functional versatility [2].

This category includes interpenetrating networks and composite systems that seek to optimize properties such as elasticity, stimulus response, or encapsulation capacity [12]. Synthesis methodologies encompass either covalent grafting between dissimilar polymer types or physical/chemical network formation through mixed-component crosslinking [2]. These formulations enable the design of multifunctional systems adaptable to a wide range of applications, from controlled release systems to smart coatings in foods.

This functional classification constitutes the basis for selecting the appropriate type of hydrogel according to the context of use, considering compatibility with the active compound, environmental conditions of the final system, and technological requirements of the manufacturing process. Regardless of their classification, the manufacture of hydrogel involves the application of chemical and/or physical crosslinking mechanisms that can be assisted for simpler or more complex compounds and technologies to produce tailor-made designs depending on their intended application. The following sections present the main crosslinking mechanisms for the production of hydrogels intended for their use in food applications.

## 3. Chemical Crosslinking

Chemical crosslinking constitutes a fundamental process characterized by the establishment of interconnected covalent bonds between macromolecular chains during the polymerization of low molecular weight monomers or during the structural modification of pre-existing polymers [7]. This molecular crosslinking mechanism is carried out through the strategic incorporation of monomers into polymeric structures or through the application of specific compounds with crosslinking capacity, facilitating interactions between various functional groups such as hydroxyls, carboxyls, and amines with substances like aldehydes, among which glutaraldehyde stands out [13]. The resulting characteristics of the materials, particularly their resistance and mechanical behavior, are significantly determined by the nature and distribution of functional groups present in the crosslinking agents employed [9]. The process culminates in a stable three-dimensional molecular architecture where the formed covalent bonds prevent both dilution and disintegration of the polymeric matrix [14].

Currently, there is a growing trend toward the implementation of crosslinking agents derived from natural sources such as genipin, plant-origin compounds like citric acid, tannic acid, and vanillin, as well as polyphenol derivatives like epigallocatechin gallate [15]. Additionally, a wide range of crosslinking agents are used to induce chemical reactions with components of polymeric and biopolymeric materials, including glyoxal, various modified dialdehydes (such as derivatives of starch, chitosan, and alginate), squaric acid, or reactive combinations like the mixture of 1-ethyl-3-(3-dimethylaminopropyl) carbodiimide hydrochloride with N-hydroxysuccinimide [16]. These crosslinking agents function as reactive elements capable of linking two or more polymeric chains, generating permanent three-dimensional structures, with both the concentration of said agents and reaction time being the main determining factors in this process [17]. Within these methods, there are in turn subcategories worth mentioning.

### 3.1. Chemical Crosslinking by Radiation

**Figure 1:**
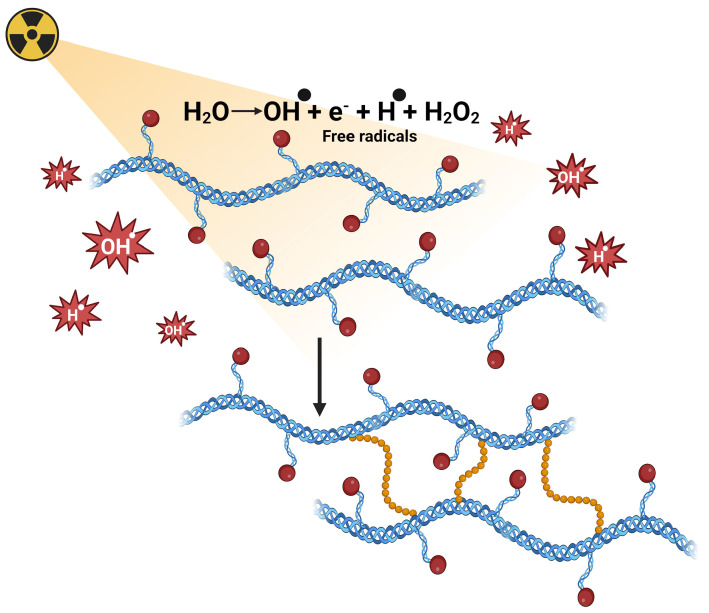
Radiation crosslinking mechanism in polymeric hydrogels: water radiolysis, free radical formation (OH• and H•), and development of three-dimensional crosslinked networks.

Chemical crosslinking by radiation represents an innovative method for hydrogel synthesis that has gained considerable attention in recent decades. This process is based on the formation of three-dimensional networks from linear polymeric chains through the use of ionizing radiation [18]. Unlike traditional chemical methods, this technique employs energy sources such as electron beams, gamma radiation, and X-rays to initiate crosslinking reactions [19]. The underlying mechanism involves the interaction of electromagnetic radiation with the polymeric material, which results in the scission of carbon-hydrogen bonds and the subsequent formation of free radicals in the macromolecular chains [20]. Simultaneously, water molecules present in the system undergo radiolysis, a process that generates highly reactive hydroxyl radicals. These radicals, together with the hydrogen radicals produced, rapidly interact with the polymeric chains to form macroradicals [21]. Figure 1 schematically illustrates the complete radiation crosslinking mechanism, from the initial bond scission to macroradical formation.

The final stage of the process occurs when these macroradicals undergo recombination reactions between different polymeric chains, establishing permanent covalent bonds that give rise to the crosslinked structure characteristic of hydrogels [22]. This methodology offers significant advantages in terms of process control, as it allows modulation of the degree of crosslinking simply by adjusting the exposure time and absorbed radiation dose, eliminating the need for additional chemical catalysts [20]. The resulting hydrogels exhibit exceptional versatility and controlled functionality, characteristics that make them especially attractive for biomedical, pharmaceutical, food, and agricultural applications [22].

### 3.2. Chemical Crosslinking by Photo-Crosslinking

Photo-crosslinking represents an advanced molecular engineering strategy that allows the construction of three-dimensional polymeric networks through controlled activation of radical polymerization processes. This methodology is characterized by the transformation of liquid precursors into stable solid matrices, leveraging the capacity of certain photosensitive compounds to generate reactive species when exposed to specific electromagnetic radiation. The versatility of the process lies in its ability to operate under moderate environmental conditions, using both ultraviolet radiation in the range of 200–400 nm and visible light between 400 and 800 nm to activate polymerization mechanisms [23,24].

**Figure 2:**
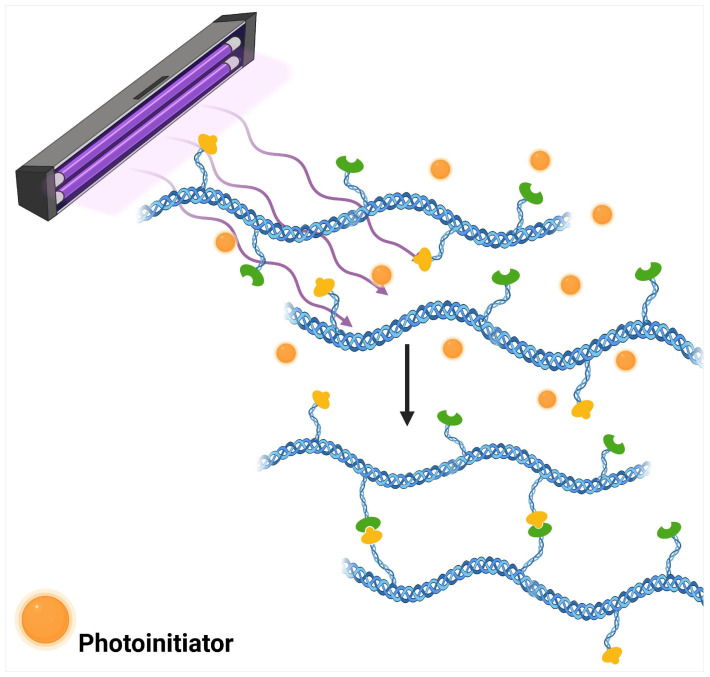
Photo-crosslinking mechanism showing UV light activation, photoinitiator decomposition into reactive radicals, and subsequent formation of covalent crosslinked polymer networks.

The scientific foundation of the process is based on the strategic incorporation of monomers and oligomers that possess unsaturated functional groups, particularly those derived from acrylic and vinylic structures, within the precursor formulation. The presence of specialized photoinitiators is essential for the success of the process, as these compounds act as mediators that absorb incident photonic energy and convert it into useful chemical energy for the initiation of chain reactions [25]. During irradiation, these photosensitive agents undergo homolytic decomposition, releasing radical fragments that subsequently interact with reactive sites of the prepolymers to establish permanent intermolecular connections [26]. This complete sequence, from light absorption to network formation, is illustrated in Figure 2.

These controlled intermolecular connections enable the design of materials with precisely tailored properties, opening diverse technological applications across industrial sectors. Particularly in food safety and environmental challenges, ref. [27] demonstrate that UV irradiation represents an effective and green technology for developing edible films with enhanced physicochemical and antimicrobial properties.

### 3.3. Crosslinking by Click Chemistry

Chemical crosslinking of hydrogels by click chemistry constitutes a molecular crosslinking method that employs highly specific reactions to establish permanent covalent bonds between polymeric chains, thus generating hydrophilic three-dimensional networks with controlled mechanical and functional properties. This gelification procedure is characterized by utilizing chemical transformations that proceed with high efficiency under normal environmental conditions, dispensing with complex external factors such as extreme temperatures or specialized atmospheres, which allows obtaining materials with superior yields and minimal contamination by residual by-products [28,29]. The methodology is distinguished by its capacity to generate crosslinks through diverse and complementary chemical mechanisms, such as azide–alkyne cycloaddition processes, Diels-Alder cycloadditive reactions between furan-maleimide systems, and thiolene addition transformations, each providing alternative synthetic routes that adapt to different molecular architectures and specific structural requirements [14]. Cycloaddition reactions constitute chemical transformations where molecular components with multiple bonds combine to generate ring structures through coordinated processes that redistribute electronic connectivity, representing one of the most employed pericyclic strategies in chemical synthesis due to their efficiency and predictability [30]. The inherent selectivity of these reactions, combined with their operational robustness and compatibility with multiple functional groups, establishes a versatile methodological framework for the construction of hydrogels with predesigned physicochemical characteristics [31].

**Figure 3:**
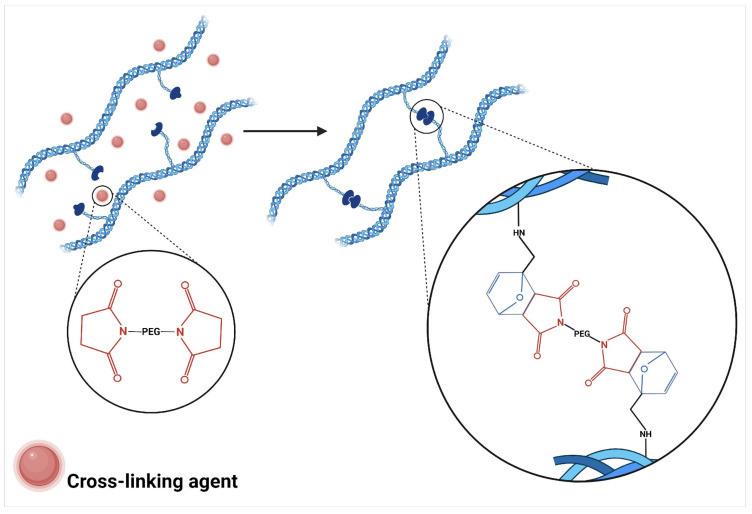
Hydrogel crosslinking mechanism through furan-maleimide Diels-Alder reaction.

On the other hand, chemical crosslinking of hydrogels by Diels-Alder reaction is a crosslinking method that utilizes [4+2] cycloaddition between diene and dienophile groups incorporated into polymeric chains to form stable covalent bonds that confer three-dimensional structure to the hydrogel [32]. This process is characterized by its chemical selectivity, as it proceeds spontaneously under physiological conditions through a concerted mechanism that does not generate byproducts nor requires external catalysts [28]. The reaction typically involves the interaction between components such as furan-maleimide, establishing specific crosslinking points that allow control of the mechanical and swelling properties of the resulting hydrogel (Figure 3) [31].

**Figure 4:**
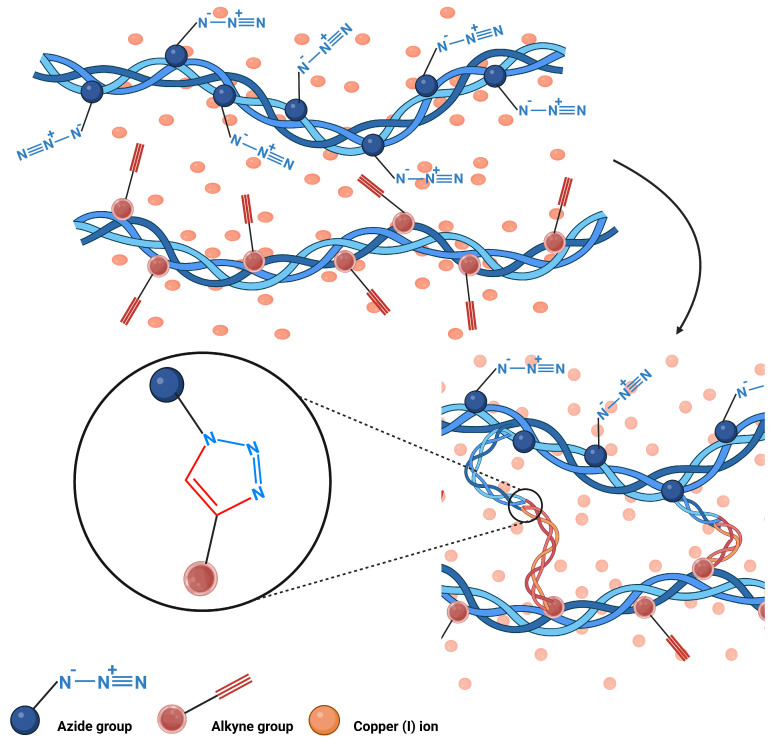
Hydrogel formation through CuAAC click chemistry between azide and alkyne-functionalized polymeric chains.

The Copper-catalyzed azide–alkyne cycloaddition (CuAAC) constitutes a fundamental synthetic methodology that involves the chemical union between an organic azide and a terminal alkyne through the mediation of a copper catalyst, resulting in the specific formation of the 1,4-disubstituted-1,2,3-triazole isomer [33]. This chemical transformation represents the paradigmatic reaction of the so-called “Click Chemistry,” characterized by its insensitivity to the electronic effects of substituents present in both reactants and its capacity to develop efficiently in diverse solvent systems, even in the absence of organic co-solvents [34]. The fundamental role of the copper catalyst is evidenced by the dramatic increase in reaction kinetics, where the presence of cupric ions accelerates the process by seven orders of magnitude compared to the non-catalyzed reaction, transforming an initially slow and poorly selective transformation into a highly regioselective process that exclusively favors the formation of the 1,4-disubstituted product [29]. The complete reaction mechanism and the regioselective formation of the 1,4-triazole product are illustrated in Figure 4.

However, despite its synthetic advantages, CuAAC presents notable biocompatibility concerns due to the use of copper ions. Residual Cu(I) can catalyze the formation of reactive oxygen species (ROS), potentially inducing oxidative stress and damage to surrounding biomolecules [35]. This has led to increasing scrutiny regarding its safety profile, particularly in contexts involving direct biological contact or ingestion. In food applications or edible hydrogel systems, the use of copper-based catalysts poses a critical limitation. Residual copper not only raises toxicity concerns but may also compromise the oxidative stability of sensitive bioactives encapsulated within the matrix. Thus, while CuAAC is a well-established method for hydrogel fabrication in biomedical research, its use in food-contact materials is highly constrained unless efficient copper-free alternatives—such as strain-promoted azide–alkyne cycloaddition (SPAAC)—are implemented [36]. Accordingly, the adaptation of click chemistry strategies for food-grade hydrogel design should prioritize catalyst-free or biocompatible crosslinking methods, ensuring both structural performance and consumer safety.

## 4. Physical Crosslinking

Physical crosslinking constitutes a process through which three-dimensional polymeric networks are generated without the intervention of external chemical agents. This phenomenon, also termed hydrogel self-assembly, emerges as a response to various environmental stimuli, among which temperature variations, pH modifications, and alterations in solvent composition stand out [12]. The underlying mechanism involves the modification of specific intramolecular forces, such as hydrophobic interactions, hydrogen bonds, and electrostatic ionic attractions, which promote the crosslinking necessary for hydrogel formation, presenting notable environmental compatibility compared to conventional chemical methodologies [37]. A distinctive characteristic of these systems lies in their reversible nature, attributable to the presence of temporary unions that can be destabilized through minimal perturbations in reaction conditions or the application of mechanical stresses [38]. The resulting structures are sustained through molecular entanglements and diverse physicochemical interactions, including charge condensation and supramolecular chemistry principles [39]. It should be noted that in the configuration of these hydrogels, complementary physical processes such as molecular association, aggregation, crystallization, and complex formation intervene, whose connections can be easily interrupted by altering environmental conditions, which is why they are frequently known as “reversible gels” [2].

### 4.1. Hydrogen Bonds

**Figure 5:**
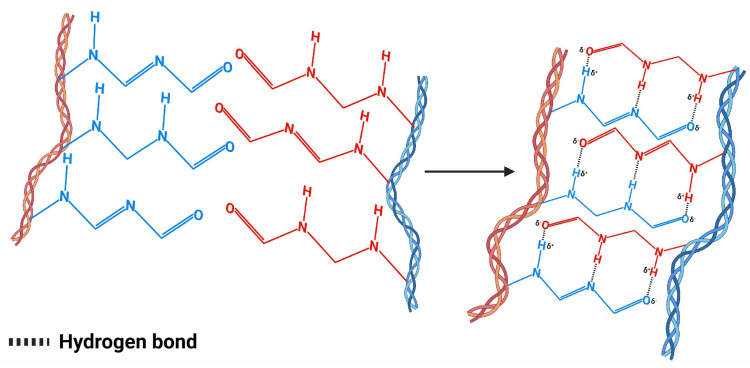
Physical crosslinking of hydrogels via multiple hydrogen bonds between electronegative atoms and hydrogen donors. Adapted from [44].

Hydrogen bonds constitute a specific type of electrostatic interaction that is established between a proton bonded to a highly electronegative atom (such as nitrogen or oxygen) and the lone electron pairs present in electronegative atoms such as nitrogen, oxygen, or fluorine [17]. This interaction is commonly represented through the notation DH···A, where D symbolizes the main electronegative atom. Due to the unequal distribution of negative charges in the electronegative atom and partial positive charges in hydrogen, the conditions for these fundamental electrostatic interactions are generated [40]. In their chemical nature, this phenomenon configures a particular dipole–dipole interaction that occurs between hydrogen covalently bonded to an electronegative atom and another electronegative atom that possesses an available electron pair, thus positioning itself at an intermediate point between weak non-covalent bonds and strong covalent bonds [41]. Regarding their bonding strength, these bridges present an energy of approximately 42 kJ mol^−1^, which places them above conventional intermolecular forces, although significantly below robust ionic and covalent bonds [42]. Figure 5 illustrates the hydrogen bonding mechanism and the cooperative network formation in hydrogel matrices. Their relevance in hydrogel structure lies in the remarkable cooperative effect they develop, since when multiple hydrogen bonds form consecutively within the polymeric matrix, their combined strength exceeds the mere sum of individual contributions, significantly contributing to the mechanical properties that characterize these biomaterials [43].

### 4.2. Hydrophobic Interactions

**Figure 6:**
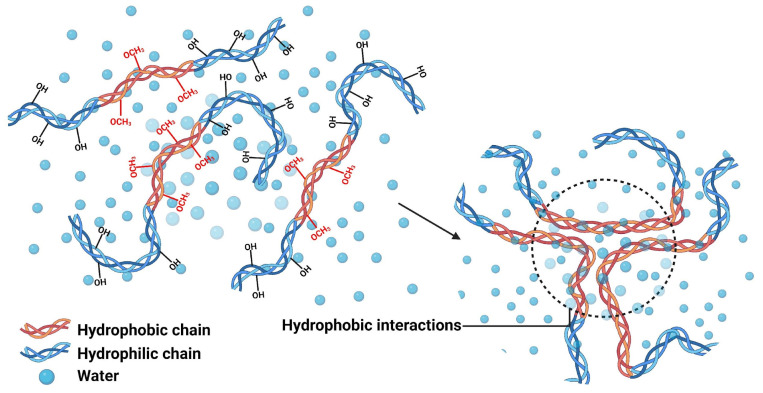
Hydrophobic interaction-mediated gelation: transition from dispersed hydrophobic chains to aggregated crosslinking nodes in hydrophilic matrices.

Crosslinking through hydrophobic forces emerges as a crosslinking strategy that exploits the energetically favorable tendency of apolar molecules to minimize their contact with aqueous media, establishing a gelification mechanism that differs fundamentally from traditional covalent approaches. This process involves the spontaneous reorganization of hydrophobic segments distributed in hydrophilic polymeric matrices, where the minimization of the system’s free energy drives the formation of aggregation domains that function as temporal and reversible crosslinking nodes, as illustrated in Figure 6 [45,46]. The efficiency of the mechanism is amplified when micellar systems actively participate in the gel architecture, generating optimized interfaces where surfactant-monomer interactions create homogeneous distributions of crosslinking points that improve the material’s mechanical response under tension [47]. The supramolecular nature of these networks confers significant operational advantages, including self-healing capabilities and structural adaptability, characteristics that position this methodology as a viable alternative for applications where the permanence of chemical crosslinking proves limiting [48].

### 4.3. Enzymatic Crosslinking

**Figure 7:**
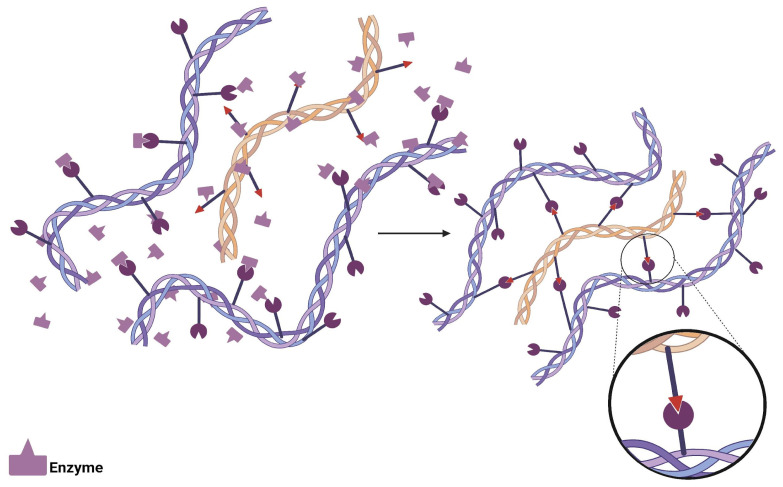
Enzyme-catalyzed crosslinking mechanism showing the specific substrate recognition and covalent bond formation between functionalized polymeric chains in hydrogel matrices.

Enzymatic crosslinking constitutes an advanced biotechnological process through which specific biological catalysts are employed to establish covalent bonds between polymeric chains, generating hydrophilic three-dimensional structures known as hydrogels. This mechanism is based on the utilization of enzymes as crosslinking agents that facilitate the formation of strong covalent bonds between polymeric chains under mild experimental conditions, which are less aggressive for sensitive components such as encapsulated drugs, therapeutic proteins, and viable cells [49]. The process is characterized by the high substrate specificity exhibited by the employed enzymes, which allows significant minimization of undesired side reactions during the crosslinking process, providing precise control over process kinetics and overall gelification rate [39]. The resulting hydrogels are formed through conjugation reactions catalyzed by specific enzymes selected according to the characteristics of the base polymer used, with transglutaminases, laccases, and peroxidases being the three main enzymatic categories employed, each with particular catalytic crosslinking modes, as shown in Figure 7 [50,51]. This enzymatic specificity represents a considerable advantage, as it allows avoidance of cytotoxic effects and ensures that certain enzymes react exclusively with specific polymers without generating cellular toxicity, resulting in rapid gelification that produces hydrogels with superior mechanical properties controllable through modulation of enzymatic activity and concentration [50]. Hydrogels obtained through enzymatic crosslinking exhibit characteristics that mimic natural extracellular matrices, manifesting distinctive physicochemical properties and functionalities that include high water retention capacity, controlled biodegradability, exceptional biocompatibility, biostability, bioactivity, optoelectronic properties, self-healing capacity, and shape memory, in addition to allowing degradation processes and morphological modifications directed by specific enzymatic mechanisms [39,52].

#### 4.3.1. Transglutaminases

Transglutaminases represent a specialized enzymatic group whose main function lies in establishing covalent connections between molecules, performing as effective crosslinking agents in multiple biological contexts and technological applications. In the animal kingdom, this enzymatic system comprises a collection of nine distinct variants that operate through a uniform catalytic mechanism centered on acyl group transfer processes [50]. The characteristic enzymatic activity involves the mobilization of glutaminic components, where the γ-carboxamide group associated with glutamine residues functions as a donor element, establishing interactions with the ε-amino group present in lysine residues that acts as an acceptor element, culminating in the establishment of ε-(γ glutamine) lysine isopeptide bonds that can develop within the same molecule or between different protein entities [53]. This catalytic capacity demonstrates particular relevance in direct crosslinking processes, especially during the generation of hydrogel structures, where the enzyme drives the development of durable isopeptide connections between primary amino groups and glutaminic carboxamide fractions integrated into polymeric chains [54]. The functional versatility of this enzymatic system extends toward the creation of molecular conjugates between protein components and aminated polysaccharides, including materials such as arabic gum and chitosan, through the establishment of new peptide connections that significantly expand their potential as a crosslinking element in biocompatible matrices [51].

#### 4.3.2. Laccases

In the field of enzymatic biochemistry, laccases represent a set of biocatalysts characterized by incorporating copper ions as essential cofactors for their functioning. These enzymatic systems are naturally present in multiple living organisms, ranging from microorganisms such as fungi and bacteria to higher organisms such as plants and insects [52]. The mechanism of action of these enzymes focuses on facilitating oxidation processes that involve the transfer of a single electron, which positions them as fundamental biological tools for establishing covalent connections between macromolecules [55]. Their crosslinking function emerges from their ability to chemically modify specific amino acid residues, particularly those containing aromatic rings such as tyrosine, transforming them into highly reactive radical species [56]. These intermediate species possess the capacity to form stable chemical bonds with other similar molecules or with functional groups available in polypeptide chains, thus establishing complex three-dimensional networks. The process culminates through the complete reduction in atmospheric oxygen, consuming four electrons in total and generating water as the final product, while simultaneously consolidating the crosslinked structures [52].

#### 4.3.3. Peroxidases

In the context of materials bioengineering, peroxidases represent fundamental biocatalysts for molecular crosslinking processes, leveraging their oxidative mechanism to form stable polymeric networks [52]. Their functioning is based on the reduction of hydrogen peroxide while extracting electrons from organic molecules, generating radical species that subsequently establish covalent bonds between polymeric chains [55]. This enzymatic family exhibits exceptional catalytic versatility, being capable of processing substrates as diverse as natural polysaccharides (chitosan, alginate, dextran), proteins (gelatin), glycosaminoglycans (hyaluronic acid), and synthetic polymers such as cellulosic derivatives and alkylene oxide copolymers [53]. The operational advantages of these biocatalysts include their non-cytotoxic profile, the capacity to perform in situ crosslinking and, particularly notable, their accelerated polymerization kinetics that allows hydrogel formation in extremely short times [52]. This combination of biocompatibility, temporal efficiency, and broad substrate spectrum positions peroxidases as strategic enzymatic tools for the development of crosslinked biomaterials [55].

## 5. Ultrasound-Assisted Crosslinking

The application of ultrasonic waves constitutes an innovative strategy that facilitates the formation of three-dimensional networks in hydrogel precursor systems through two main molecular activation pathways. On one hand, ultrasonic energy induces water fragmentation during cavitation events, releasing highly reactive species that act as natural catalysts of the crosslinking process, thus eliminating dependence on external chemical additives and allowing more precise control over gelification kinetics [57,58,59]. Simultaneously, in protein-based systems, ultrasonic vibrations promote conformational changes that destabilize native molecular architectures, exposing previously hidden reactive sites and facilitating intermolecular interactions that strengthen the hydrogel matrix [31].

This dual approach demonstrates particular effectiveness in whey proteins, where induced structural modifications increase the availability of reactive groups essential for the formation of stable covalent bonds, resulting in matrices with superior mechanical and functional properties [60]. The versatility of this technology extends further to its capacity to enhance complementary enzymatic processes, such as transglutaminase-catalyzed reactions, where ultrasonic pretreatment significantly optimizes the final crosslinking degree and characteristics of the resulting hydrogel [61].

Despite its promise as a green and non-chemical crosslinking method, the industrial adoption of ultrasound-assisted hydrogel synthesis remains limited. One of the main challenges is the need for specialized equipment that can deliver uniform acoustic energy at large scale, especially when working with viscous biopolymer solutions or large volumes [62]. In this sense it is well known that the cavitation process effectiveness is inherently sensitive to system parameters such as temperature, viscosity, polymer concentration, and sonication frequency [63]. In addition, in terms of energy consumption, high-intensity ultrasound may lead to increased operational costs when scaled up, particularly in continuous flow systems, while not all polymer systems respond favorably to acoustic cavitation. Some of them may degrade or denature under prolonged exposure, limiting the choice of substrates for industrial formulations [64]. Nonetheless, recent studies highlight ultrasound’s potential in emulsion-based hydrogel systems, where it facilitates the dispersion and encapsulation of lipophilic bioactive compounds without the need for surfactants or chemical initiators [65]. This makes ultrasonic processing particularly attractive for clean-label food formulations and biodegradable packaging. In this regard, proper design of ultrasonic devices with adjusted frequency, wave amplitude and homogeneity delivered ultrasonic intensity with capable systems to inline monitoring of gelation parameters could enable more controlled and scalable implementations. At this point, multidisciplinary efforts between materials scientists and process engineers will be essential to translate this technology from lab-scale innovation to commercial reality.

To finally summarize what has been discussed in Section 3 respect all the pros, cons and scalability potential, Table 1 shows a comparison of the crosslinking mechanisms currently available for the manufacturing of functional hydrogels. It can be observed that each crosslinking mechanism presents specific trade-offs between efficiency, biocompatibility, and scalability. While chemical methods dominate industrial use, their toxicity and regulatory hurdles remain challenging. Ultrasound-assisted crosslinking offers a sustainable and catalyst-free alternative with strong potential for food applications, yet its industrial deployment requires further development in equipment design and process standardization.

## 6. Structural Configurations

The structural architecture of a hydrogel is key to determining its mechanical properties, swelling capacity, porosity, release rate of active compounds, and stimulus response. Depending on how polymeric chains are organized within the three-dimensional network, hydrogels can be classified into different configurations. Homopolymeric hydrogels are composed of a single type of monomer or polymer, whose network is formed through self-crosslinking. Their structural simplicity facilitates characterization but may limit affinity toward certain bioactive compounds or the possibility of adjusting multiple properties simultaneously. Examples include hydrogels based on polyacrylamide or pure alginate [2].

**Figure 8:**
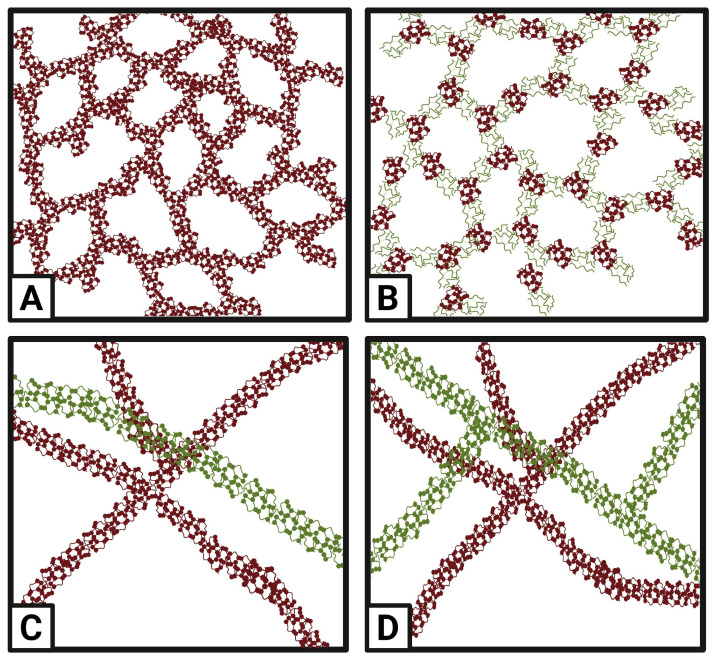
Comparative illustration of hydrogel network architectures: homopolymeric single-component system (**A**), copolymeric multi-component system (**B**), semi-IPN with one crosslinked and one linear network (**C**), and complete IPN with dual crosslinked networks (**D**). Adapted from [78].

Copolymeric hydrogels arise from the combination of two or more different monomers that polymerize together to form networks with improved or customized properties. This strategy allows incorporating specific functional domains, improving stimulus sensitivity, or increasing compatibility with biological systems. Block, grafted, or random copolymers offer versatility in structural and functional design [9]. Interpenetrating polymer networks (IPN) are formed by at least two polymeric networks intertwined but not covalently bonded. They can be: (i) Semi-IPN, in which one network is crosslinked, and the other is free, and (ii) Complete IPN, where both networks are crosslinked simultaneously. These structures combine the individual properties of each network, improving elasticity, degradation resistance, or controlled release of active ingredients. They are particularly useful in smart delivery systems, tissue regeneration, and food encapsulation [12]. As shown in Figure 8, these four fundamental configurations—homopolymeric, copolymeric, semi-IPN, and complete IPN—each present distinct network architectures. Composite or hybrid hydrogels incorporate functional fillers or particles (e.g., nanoparticles, clays, lipids) within the polymeric network to improve mechanical, bioactive, or rheological properties. Hydrogels reinforced with nanomaterials can acquire additional functionalities such as conductivity, sensitivity to magnetic fields, or antimicrobial properties [10]. Among hybrid systems, eutectic gels and bigels have emerged as promising strategies for food applications due to their multifunctionality.

Eutectic gels, formed by incorporating deep eutectic solvents (DES) into the hydrogel network, offer tunable properties such as enhanced stability, flexibility, adhesivity, and altered water-polymer interactions [66,67]. In food systems, DES can be enriched with bioactive compounds, acting as concentrated extracts or carriers that facilitate incorporation into edible formulations, bioplastics, or functional ingredients [68]. Their ability to partially or completely replace water allows precise control over gel mechanics, bioactive release, and thermal resistance, which is particularly useful for designing foods with enhanced shelf-life or functional properties. In contrast, bigels combine hydrogel and oleogel phases, enabling the simultaneous encapsulation of hydrophilic and hydrophobic compounds [69]. In food technology, bigels systems combining hydrogels and oleogels represent a promising innovation, merging the advantages of water- and oil-based structures. Bigels have been investigated as nutraceutical and antioxidant carriers, fat substitutes, and functional ingredients. For example, a bigel composed of sesame oleogel and alginate hydrogel successfully replaced fat in butter spreads, yielding a product with balanced fatty acid profile, desirable texture, and improved probiotic viability [69,70]. Similarly, a bigel with grape seed oil oleogel and alginate hydrogel was proposed as a cocoa butter substitute in chocolate, reducing saturated fat content and enhancing thermal resistance due to an increased melting point [71,72,73]. Other applications of bigels in food include delivery systems for bioactive compounds [74,75], fat substitutes in meat [76], and bakery products [77]. Each structural configuration whether homopolymeric, copolymeric, IPN, or hybrid offers unique advantages depending on the intended application. Simple homopolymers may be preferred for rapid gelification, whereas multifunctional formulations such as IPNs, eutectic gels, and bigels are more suitable for advanced applications requiring prolonged release, physiological resistance, or dual functionalities (e.g., food and therapeutic uses). Although both systems provide multifunctionality, their positioning in food applications differs. Eutectic gels are primarily used for controlled release of bioactives, stabilization of sensitive compounds, or incorporation of green solvent-based extracts. Bigels, on the other hand, are more oriented toward structuring foods, fat replacement, and simultaneous delivery of hydrophilic and lipophilic ingredients. The complementary nature of these approaches suggests potential for hybrid strategies, where DES-enriched hydrogels could be integrated into bigel systems to combine the benefits of targeted bioactive delivery with versatile food structuring. It is important to highlight that, the selection between eutectic gels and bigels in food applications depends on the target functionality: eutectic gels for chemical stabilization and bioactive enrichment, bigels for dual-phase encapsulation and textural/fat replacement purposes. Indeed, understanding these distinctions allows for more rational design of multifunctional food systems, bridging the gap between innovative materials and practical applications.

## 7. Applications in Foods

In the food industry, hydrogels represent an emerging technology to improve the stability, bioavailability, and functionality of nutrients, while also contributing to the design of structured foods and smart packaging. To illustrate the diversity of approaches in this field, Table 2 presents a comparative overview of hydrogel synthesis methodologies specifically designed for food applications, detailing the materials employed, crosslinking strategies, processing conditions, and key performance indicators.

### 7.1. Encapsulation and Delivery of Nutrients for Functional Foods

One of the most relevant applications of hydrogels is the encapsulation of sensitive nutrients such as vitamins, probiotics, antioxidants, and fatty acids, protecting them against thermal processing, oxidation, or gastrointestinal degradation [7,8]. This enables controlled release in specific intestinal regions and improves absorption. Several studies have demonstrated this efficacy with different polymeric matrices. For instance, rice flour-based hydrogels for vitamin D3 retained above 80% after 4 weeks at 40 °C [87], while alginate-copper hydrogels protecting folic acid at gastric pH and releasing it at intestinal pH through an “egg-box” structure [88]. Pectin-starch hydrogels have served to encapsulate >94% of *Lactobacillus plantarum* with pH-controlled release [89]. Other authors reported that β-lactoglobulin-albumin hydrogels achieved up to 95% riboflavin encapsulation via ionic crosslinking [90]. In the design of functional foods, hydrogels enable co-encapsulation of bioactives for personalized nutrition, with antimicrobial, immunomodulatory, or antioxidant functions [9]. For example, whey protein microgels developed by Nestlé Health Science achieved a 22% reduction in postprandial glucose in type 2 diabetes patients, attributed to enhanced bioavailability of peptides stimulating GLP-1 and insulin [91]. Antioxidant functional gels have also been achieved through methylcellulose-gelatin systems incorporating olive powder, yielding high hydroxytyrosol retention with strong sensory acceptability [92]. Such advances illustrate how hydrogel matrices protect and deliver bioactives in a controlled way, enabling functional foods tailored to specific health needs. All these examples emphasize the versatility of hydrogels as protective matrices for bioactive compounds such as polyphenols, probiotics, and lipid-soluble vitamins. At this point, a key trend is the design of pH and enzyme-responsive systems to target specific intestinal segments, improving bioavailability and minimizing nutrient degradation [93]. However, the integration of lipophilic and thermolabile compounds still poses formulation challenges, especially under industrial processing conditions. Therefore, future advances are expected to focus on multi-layered or hybrid hydrogel systems combining physical and enzymatic crosslinking for greater structural integrity and better controlled release precision.

### 7.2. Texture Modification and Functional Foods

Hydrogels are also valuable in emulsification and texturization, where protein or polysaccharide-based systems emulsify oils and form semi-solid textures similar to dairy or meat products, supporting vegan or low-fat formulations [78]. For instance, alginate emulsion hydrogels have been applied in pork burger reformulations, replacing animal fat with healthy oils while reducing saturated fat and preserving sensory quality [94]. Similarly, soy protein isolate-based emulsion hydrogels were designed as fat mimetics for plant-based salami analogues, maintaining marbling and improving sensory acceptance [95]. For soft foods, enzymatically crosslinked β-lactoglobulin hydrogels demonstrated superior softness and rheological properties compared to agar gels, highlighting their potential in jellies, puddings, and specialized diets [79]. As shown, research in this area increasingly targets the use of protein- and polysaccharide-based hydrogels to engineer novel textures, mimicking the mouthfeel and rheological behavior of fat or gluten-containing products [78]. There is growing interest in tailoring viscoelasticity and gel strength through fine control of crosslinking density and polymer interactions. Despite promising lab-scale prototypes, translating these textural innovations into scalable, clean-label formulations remains a critical gap, especially considering the sensory expectations and regulatory constraints in commercial food systems.

### 7.3. Hydrogels in Food Active Packaging

Hydrogel-based materials are emerging as multifunctional platforms for active packaging, capable of integrating antimicrobial agents, oxygen scavengers, and freshness indicators [96]. Hydrogels also play a growing role in active packaging, acting as biodegradable films or coatings capable of releasing antimicrobial agents, regulating humidity, or even detecting spoilage. For example, β-lactoglobulin hydrogels with epigallocatechin-3-gallate demonstrated antibacterial activity against *E. coli* and *S. aureus* [97]. Chitosan-PVA hydrogels reinforced with thymol-zeolite nanohybrids improved barrier properties and extended strawberry shelf life up to 21 days [98]. Smart packaging has also been developed through chitosan-polyurethane hydrogels functionalized with eugenol, showing antibacterial effects, mechanical strength, and visible pH response through color changes, making them suitable for perishable protein foods [99].

A significant research trend is the development of biodegradable, edible films that combine high water content with targeted functionality, enhancing both food preservation and environmental sustainability. However, current systems face limitations in moisture sensitivity and mechanical strength, requiring hybrid strategies such as UV-induced crosslinking or inclusion of nanofiller to meet packaging performance standards.

### 7.4. Hydrogels in Cultured Meat

A particularly innovative field is the use of hydrogels as scaffolds for cultured meat, where natural polymer-based systems provide bioactive architectures that regulate cellular behavior, support oxygen and nutrient diffusion, and remain edible and safe for human consumption. Alginate hydrogels modified with whey proteins enhanced muscle cell proliferation with tunable mechanical properties [100]. Edible whey protein hydrogels crosslinked with transglutaminase and CaCl_2_ improved wettability and zeta potential, supporting porcine stem cell adhesion and differentiation [101]. Alginate hydrogels ionically crosslinked with high Ca^2+^ concentrations improved myoblast viability [102], while collagen hydrogels with aligned microgrooves promoted directional growth and enhanced expression of muscle-specific proteins [103]. These advances highlight the evolution from simple scaffolds to bioengineered edible matrices that integrate mechanical, biochemical, and topographic cues to mimic natural muscle tissue. However, despite these promising developments, several critical challenges remain to be addressed before large-scale application of hydrogel-based scaffolds in cultured meat becomes feasible. First, the biopolymeric matrices must balance mechanical strength and elasticity with cellular permissiveness, which becomes particularly complex when scaling from 2D cultures to volumetric tissue structures. Moreover, hydrogel degradation rates must be fine-tuned to support tissue maturation without compromising edibility or sensory quality. Another limitation lies in the sourcing and cost-effectiveness of bioactive hydrogel components, especially when considering food-grade regulatory requirements. While studies using alginate, collagen, and whey proteins have demonstrated proof-of-concept, their translational viability depends on consistent reproducibility, cost-efficiency, and consumer acceptability. Future research should focus on developing modular, food-safe hydrogels that combine tunable degradation, biofunctionality, and large-scale manufacturability, potentially integrating 3D bioprinting or dynamic perfusion bioreactors to enhance structural fidelity and nutrient supply during meat tissue cultivation [100,101,102,103].

## 8. Emerging Trends and Current Challenges

**Figure 9:**
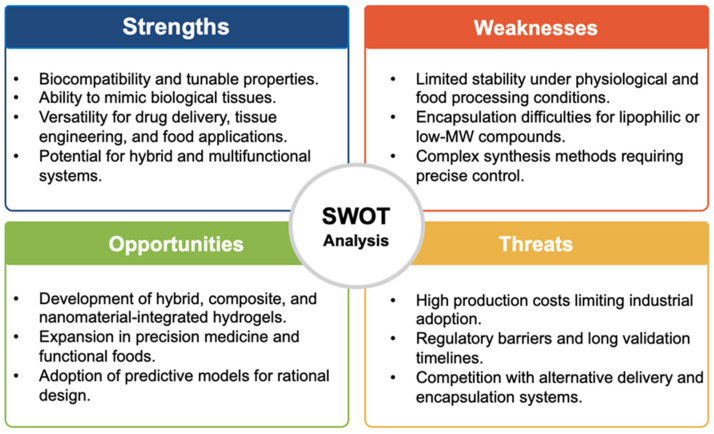
SWOT analysis of hydrogels, summarizing their main strengths, weaknesses, opportunities, and threats in the context of biomedical and food applications.

Recent advances in hydrogel research highlight several emerging directions that are expanding their applicability across biomedicine, food, and smart materials. A major trend involves the development of smart hydrogels sensitive to multiple stimuli, which respond reversibly to changes in pH, temperature, ionic strength, light, or biological signals. These systems enable controlled release and localized activation, playing a crucial role in precision medicine, targeted therapies, and personalized functional foods [38]. Another innovative approach is the design of self-assembling and self-healing hydrogels, capable of reconstituting their structure after mechanical or chemical damage. This property, generally mediated by reversible interactions such as hydrogen or ionic bonds, makes them suitable for dynamic environments, including implantable medical systems, reusable packaging, and adaptive smart coatings [37]. Finally, the combination with nanomaterials, including silver, silica, graphene nanoparticles, lipids, or other active agents has enabled the creation of composite hydrogels with novel functionalities. Despite their versatility and potential, hydrogels face important challenges that limit their large-scale adoption in food, biomedical, and technological applications. A major concern is stability and functionality under physiological conditions, as these materials must resist harsh environments such as gastric acidity, enzymatic activity, and fluctuating temperatures without compromising their biocompatibility. This demands the design of stronger yet safe polymeric networks [7]. Another limitation is related to encapsulation capacity, since hydrogels often struggle to efficiently incorporate and protect lipophilic or low molecular weight compounds. Addressing this issue requires advances in hybrid systems, co-encapsulation strategies, and innovative crosslinking methods that can expand their versatility [9]. From a technological standpoint, industrial scalability and reproducibility remain key barriers. The transition from laboratory prototypes to mass production involves specialized equipment, stringent control of synthesis conditions, and high manufacturing costs. These factors slow down the integration of hydrogels into regulated sectors such as food and pharmaceutical industries [78]. In addition, regulatory and validation requirements represent another critical obstacle. To ensure safety, efficacy, and long-term stability, extensive preclinical and clinical studies are necessary, as well as compliance with strict regulatory frameworks. These steps, while essential, can significantly delay commercialization (Figure 9).

Despite the remarkable progress in hydrogel research, significant challenges remain regarding their stability, encapsulation efficiency, large-scale production, and regulatory approval, and overcoming these limitations requires a multidisciplinary approach that combines expertise in chemistry, materials and food science, and biotechnology. Addressing these challenges will depend on advances in material design, hybridization strategies, and predictive modeling, as well as stronger academia–industry collaborations, which together will be crucial to accelerate the development of safe, functional, and scalable hydrogel technologies and translate them into reliable, economically viable solutions for biomedical and food applications.

## 9. Conclusions

The development of polymer-based hydrogels for food and biotechnological applications has advanced rapidly, with significant progress in tailoring structural, mechanical, and functional properties to meet specific needs. Current research demonstrates that hydrogels can be engineered not only as carriers of bioactive compounds, scaffolds for cell growth, or controlled release matrices, but also as active components that contribute to food stability, safety, and health promotion. However, several challenges remain. (i) First, the rational design of polymer networks still requires deeper understanding of the relationship between molecular structure, crosslinking strategies, and functional performance under realistic processing and storage conditions. In particular, scaling laboratory results to industrially relevant systems remains a critical barrier, while (ii) issues of biocompatibility, regulatory approval, and consumer perception must be addressed to ensure successful translation of hydrogel technologies into the food sector.

Looking forward, emerging opportunities lie in the following areas:

− Smart and responsive hydrogels that adapt their behavior to pH, temperature, or enzymatic activity, enabling precision delivery of nutrients or bioactives.
− Green and sustainable synthesis routes, employing renewable biopolymers and mild processing conditions to minimize environmental impact.
− Integration with advanced processing technologies (e.g., 3D printing, microfluidics, and membrane-based systems) to design customizable food structures and functional biomaterials.

Finally, the multi-functional biorefinery approaches, where hydrogels can be obtained from agro-industrial by-products, linking waste valorization with added-value functional ingredients. In this context, hydrogel research is moving beyond proofs of concepts and towards multidisciplinary innovation, requiring collaboration between polymer scientists, food technologists, and biotechnologists. Strengthening this interface will be essential to unlock the full potential of hydrogels as sustainable and versatile materials for the next generation of functional foods and biotechnological applications.

## Figures and Tables

**Table 1:** Comparative Analysis of Crosslinking Mechanisms for Functional Hydrogels.

Crosslinking Mechanism	Reaction Conditions	Gelation Speed	Mechanical Strength	Biocompatibility/Regulation	Encapsulation of Lipophilic Compounds	Industrial Scalability	References
Chemical (e.g., CuAAC, UV, aldehydes)	May require catalysts or harsh conditions (pH, temperature, radiation)	Fast (seconds to minutes)	High (covalent bonding)	Variable (may require purification of toxic residues)	Limited (weak interactions with nonpolar molecules)	High, but process-dependent (e.g., photochemical or thermal methods)	[14,16,17,49]
Physical (e.g., H-bonds, ionic, hydrophobic)	Mild, no need for toxic reagents	Moderate to fast	Low to medium (reversible interactions)	High (generally good biocompatibility)	Low to moderate (rapid diffusion and poor stability)	High (simple and reproducible processes)	[37,39,50]
Enzymatic (e.g., TGase, laccase, peroxidase)	Mild, aqueous, physiologically relevant conditions	Moderate (minutes to hours)	Medium to high (specific covalent bonding)	Very high (GRAS enzymes, no toxic residues)	Limited (polar environments restrict lipophilic interactions)	Medium (cost and enzyme availability can be limiting factors)	[50,52]
Ultrasonic (acoustic cavitation-induced)	Generally mild, but requires specific equipment	Very fast (seconds)	Variable (depends on polymer system)	High (no external reagents required)	Promising (enables direct emulsification of lipophilic compounds)	Medium to low (emerging technology, needs validation)	[57,58,59,61]

**Table 2:** Summary of the main applications of hydrogels in food materials.

Substrate/ Base Material	Reported Limitations	Identified Advantages	Main Results	Operating Specifications	Crosslinking Mechanism	References
Beta-lactoglobulin (β-LG)	Concentrations < 5% do not gel; Two-stage process with extended times; Lower mechanical resistance vs. conventional gels	Low protein concentration required (5%); Green process without hazardous chemicals; Superior texture: softer and less brittle than agar	G’: <10^3^ Pa; Rupture force: 0.40 ± 0.08 N; Rupture penetration: 3.7 ± 0.3 mm; Morphology: aggregates 50–150 × 10 nm; Thermal stability: 329.76 °C	pH 7.5 (Tris-HCl 50 mM), 80 °C × 30 min (Heat treatment), 37 °C, 300 rpm, ON (Incubation), 80 °C × 30 min (Inactivation), 4 °C (Storage)	Thermal + Enzymatic	[79]
Whey protein 8.8% + Sodium alginate 2.4% (80/20 *w*/*w* ratio)	Rapid release, pH dependence, coating loss, ionic sensitivity	Biocompatible, gastro-resistant, controlled release, simple process	EE: 23–100%, size: ~1.5 mm, coating: 68–146 µm, release: 5–80%/h	pH 7.0, denaturation 80 °C/40 min, extrusion 23G needle, coating 5 min	Thermal + Ionic	[80]
Polygalacturonic acid (polyGalA)–anionic polysaccharide derived from pectin	Slower release in complete hydrogels vs. sectioned; Limited diffusion by mesh size and electrostatic repulsions	Dual control of dosage and release rate; Ideal candidates for oral release systems; Maintenance of native protein form	Release by Fickean diffusion (*n* ≈ 0.5); Mesh size: 75–95 Å; Nearly complete recovery of nominal BLG/polyGalA ratio	pH: 7.0 ± 0.3; [NaCl]: 10 mM; Gelation temperature: ambient; Gelation time: 24 h; [polyGalA]: 10–20 g/L; [BLG]: 10–16 g/L; Release temperature: 37 °C	Ionic	[81]
Sodium alginate + Whey protein nanofibers	Decrease in properties >50 mg/mL WPN, Requires more in vivo release studies	Cold processing preserves thermosensitive compounds, Superior mechanical properties, Excellent bioactive encapsulation	G’ = 768.2 Pa (3.75× greater than pure SA), Hardness = 273.3 g (2.26× greater), Curcumin encapsulation = 91.6%	25 °C, pH 6.5, 12 h of gelation	Ionic with Ca^2+^ + electrostatic and hydrogen interactions	[82]
Sodium alginate + β-lactoglobulin	Process sensitivity: tail formation under suboptimal conditions. Critical concentrations	Improved thermal stability. UV protection: 90% reduction in photodegradation. Preferential targeted release in intestinal environment. Preservation of antioxidant activity (DPPH, ABTS, FRAP)	Encapsulation efficiency: 84.58%. Average size: 1053.73 nm. Zeta potential: −25.44 mV. Intestinal release: 84% vs. 36% (free polyphenols). Bioaccessibility: 3× superior to free polyphenols	Vibratory frequency: 953.14 Hz. Voltage: 1893.76 V. Temperature: 45 °C. pH: 4.6	Ionic with Ca^2+^	[83]
β-lactoglobulin + sodium alginate	Secondary structure loss (20%), conformational changes	No pretreatment, mild conditions, high efficiency	>70% crosslinking, polymers 18–240 kDa, hydrophobic interaction with alginate	pH 8.5, 37 °C, 24 h, 300 rpm	Enzymatic + Ionic	[84]
β-lactoglobulin	Intramolecular crosslinking, lower polymerization	Rapid reaction, structural preservation	Polymers 18–120 kDa, 37 crosslinks, preserved native structure	pH 8.5, ambient temperature, 30 min	Covalent bonds	[84]
Thiolated tetrafunctional polyethylene glycol (PEG-SH)	Cell viability: Only 28.8–30.8% post-degradation with L-cysteine; Toxicity: Risk of cell reduction by H_2_O_2_; Technical: Difficulty applying standard rheological criteria for rapid reactions	Mild conditions compatible with biological materials; No UV light exposure required; Ambient temperature operation; Physiological pH (7.3)	Crosslinking time (tcross): 2–12 min, Equilibrium storage modulus (G’eq): 1–16 kPa	pH 7.3 (DPBS), 25 °C × 1 h (Gelation)	Enzymatic	[49]
Gelatin methacrylate (GelMA) derived from type A collagen	Decreased strength with prolonged exposure, lower swelling vs. pure GelMA, brittleness at high concentrations	Superelasticity, spontaneous self-healing, water-resistant adhesion, excellent biocompatibility, surgical application without sutures, sensory capability with CNTs	Maximum strength: +4.3×, Compressive modulus: +2.5×, Elongation: +6× (277%), Adhesion: 81 kPa, Recovery: 90%	GelMA: 10–20% (*w*/*v*), Temperature: 37 °C, Time TA: 24 h optimal, Concentration TA: 100% optimal.	Dual: chemical covalent (primary) and physical by hydrogen bonds (secondary)	[85]
Propylene glycol alginate (PGA) + β-lactoglobulin nanoparticles	Use of ethanol, loss in intestinal fluid, pH dependence, not evaluated in vivo	Green method, biocompatible, dual protection, controlled release, excellent stability	LGG encapsulation > 98%, curcumin retention 91.3% (4 weeks), LGG survival 9.72 log CFU mL^−1^	pH 4.0, ambient temperature, 12 h of gelation	Physical non-covalent induced by solvent	[86]

## Data Availability

No new data were created or analyzed in this study. Data sharing is not applicable to this article.
